# Machine learning-driven PET-CT and clinical pathology model for predicting mediastinal lymph node metastasis in non-small cell lung cancer: a retrospective cohort study

**DOI:** 10.7717/peerj.20788

**Published:** 2026-02-03

**Authors:** Taiyu Bi, Min Qiang, Xiaotian Duan, Yipeng Yin, Wenyu Zhang, Zhe Chen, Xinjun Zhang, Jianzun Ma, Bowei Zhang, Mingbo Tang, Wei Liu

**Affiliations:** Department of Thoracic Surgery, The First Hospital of Jilin University, Changchun, China

**Keywords:** NSCLC, Mediastinal lymph node metastasis, PET-CT, Machine learning, XGBoost, Predictive model

## Abstract

**Objective:**

This study aims to evaluate whether Positron Emission Tomography–Computed Tomography (PET-CT) imaging features of primary tumors and lymph nodes, combined with clinical and pathological data, can accurately predict mediastinal lymph node metastasis (MLNM) in resectable non-small cell lung cancer (NSCLC) using machine learning models.

**Methods:**

A retrospective study was conducted on 390 NSCLC patients who underwent tumor resection and lymph node dissection between January 2017 and December 2023. All patients received 18F-fluorodeoxyglucose (18F-FDG) PET-CT scans within two weeks before surgery. Data from 390 primary tumors and 1,026 lymph node stations were analyzed. Clinical and PET-CT imaging features were extracted, and feature selection was performed using a random forest algorithm. Eight machine learning models were evaluated, including Logistic Regression, classification and regression tree (CART), support vector machine (SVM), gradient boosting decision tree (GBDT), Random Forest, multi-layer perceptron (MLP), extreme gradient boosting tree (XGBoost) and k-nearest neighbor algorithm (KNN).

**Three models were developed:**

Tumor-Pathology-Clinical (TPC), Lymph-Pathology-Clinical (LPC), and Tumor-Lymph-Pathology-Clinical (TLPC). Model performance was assessed using Receiver Operating Characteristic (ROC) curves, Decision Curve Analysis (DCA), and confusion matrices.

**Results:**

The TLPC model, based on the XGBoost algorithm, showed the best performance, with an Area Under the Curve (AUC) of 0.90 (95% CI [0.883–0.957]), specificity of 0.84, and sensitivity of 0.96 (*P* = 0.0069; significant at *P* < 0.05). In comparison, the TPC model achieved an AUC of 0.67 (95% CI [0.647–0.703]), specificity of 0.46, and sensitivity of 0.56 (*P* = 0.7037; not significant). The LPC model showed intermediate performance, with an AUC of 0.78 (95% CI [0.713–0.751]), specificity of 0.73, and sensitivity of 0.84 (*P* = 0.0269; significant at *P* < 0.05). All *P*-values were derived from DeLong’s test comparing AUCs between models, with statistical significance defined as *P* < 0.05. Of the 1,026 lymph node stations analyzed, 204 showed metastasis, while 822 did not. XGBoost consistently outperformed other models in predicting MLNM.

**Conclusion:**

Combining PET-CT imaging features of primary tumors and lymph nodes with clinical and pathological data shows promise for accurately predicting MLNM in NSCLC. The TLPC model offers a non-invasive method for identifying lymph node metastasis, supporting personalized treatment strategies. However, since PET-CT was performed selectively rather than routinely acquired, external validation across diverse clinical settings is warranted to confirm model generalizability.

## Introduction

Lung cancer remains one of the leading causes of cancer-related deaths worldwide (Martins et al., 2012). Non-small cell lung cancer (NSCLC) accounts for 85% of all lung cancer cases, with its staging primarily relying on the tumor-node-metastasis (TNM) system, where N2 lymph node metastasis is crucial for treatment planning (Rusch et al., 2009). The N2 stage refers to metastasis in the ipsilateral mediastinal lymph nodes, encompassing stations 2-9, which significantly affect patient prognosis and treatment decisions (Martins et al., 2012). Resectable NSCLC is generally defined as having a primary tumor that can be resected with no distant metastasis (M0) or the presence of local lymph node metastasis (N1-2) without distant metastasis (M0) (Delman, 2020). However, in clinical practice, patients with mediastinal lymph node metastasis (MLNM) experience significantly lower postoperative survival rates, often requiring neoadjuvant treatment before surgery or opting for conservative treatment instead. Thus, the determination of MLNM directly influences the treatment plan. Therefore, accurate preoperative diagnosis of N2 lymph node metastasis is particularly important (Yu et al., 2024).

The gold standard for MLNM in NSCLC patients is histopathological examination. Methods for confirming pathological metastases include ultrasound bronchoscope-guided transbronchoscopic needle aspiration biopsy (EBUS-TBNA) and video-assisted mediastinoscopy (VAM). However, for patients with surgically resectable NSCLC, invasive mediastinal lymph node examination before surgery also has its drawbacks, including false negative results due to incorrect lymph node selection, insufficient samples due to technical problems, inability of patients to withstand invasive biopsy, and possible complications such as bleeding, infection or neurovascular injury (Wang et al., 2023). Fortunately, the non-invasive 18F-fluorodeoxyglucose (18F-FDG) Positron Emission Tomography–Computed Tomography (PET-CT) is able to provide both metabolic and anatomical information about lymph nodes, showing great potential for predicting lymph node metastasis in many cancer types, including NSCLC. According to established PET-CT diagnostic criteria, lymph node metastasis was defined by a short-axis diameter of ≥1 cm and/or an SUVmax ≥ 2.5, thresholds that have been shown to yield sensitivities of 80–90% and specificities of 85–95% for detecting metastatic lymph nodes in NSCLC (Nakanishi et al., 2021). These parameters were adopted as the baseline diagnostic thresholds for identifying potential metastatic lymph nodes in this study (Bauwens et al., 2008; Park et al., 2010). In addition, tumor load indicators such as total focal glycolysis (TLG) and metabolic tumor volume (MTV) can also be used to predict lymph node metastasis (Liao et al., 2023). However, imaging features in the diagnosis of MLNM may be biased by different study populations and sample sizes, so larger studies are needed to verify this.

Previous studies on clinical risk factors for MLNM have indicated that risk factors include a history of smoking (Na et al., 2011), elevated tumor markers such as carcinoembryonic antigen (CEA) and squamous cell carcinoma antigen (SCC), and age over 60 (Huang et al., 2023), among others. Although there are many types of lung cancer tumor markers, their specificity and sensitivity are relatively weak, and their diagnostic value for MLNM is still under exploration. Other clinical characteristics, such as smoking, alcohol consumption, coronary heart disease, hypertension, and type 2 diabetes, have shown inconsistent results across different studies, and no definitive conclusions have been reached (Chen et al., 2020; Honguero Martinez et al., 2016; Zeng et al., 2022). Further research is needed to explore the clinical factors leading to MLNM.

Traditional statistical methods typically perform univariate analysis followed by logistic regression to screen for risk factors, subsequently establishing a visual clinical prediction model and evaluating it (Chen et al., 2022; Lv et al., 2021; Mattes et al., 2015; Qi et al., 2021; Qiao et al., 2022). However, this analysis requires converting continuous variables into categorical ones, which may lead to an increase in positive results.

Such dichotomization can distort the underlying relationships by reducing information and introducing arbitrary thresholds, which may inflate apparent associations or, conversely, obscure true ones depending on the data structure. Additionally, because logistic regression assumes a linear relationship between predictors and the outcome, it is particularly sensitive to multicollinearity—when predictors are highly correlated, their individual effects become unstable and may require variable exclusion or transformation. Although certain machine learning algorithms (*e.g*., tree-based models) can better tolerate collinearity, it remains a general challenge across modeling approaches, potentially distorting variable importance and interpretability if not properly addressed. Some previous studies further support the effectiveness of multimodal approaches. For instance, a study based on a support vector machine (SVM) classification method utilized radiomics analysis to predict lymph node (LN) metastasis in NSCLC, achieving an AUC of 0.81. However, unlike our study, this research did not independently model mediastinal lymph nodes (Yin et al., 2021). In contrast, Liao et al. (2023) improved the model’s predictive capability by combining clinical data and imaging features using multifactorial logistic regression, raising the area under curve (AUC) to 0.85, although it still did not reach the accuracy of the Tumor-Lymph-Pathology-Clinical (TLPC) model. This highlights the crucial importance of incorporating key clinical features of patients in building an excellent predictive model.

Regarding the selection of clinical features, it is considered that while the radiological characteristics of the primary tumor perform well in predicting lymph node metastasis (LNM) in NSCLC patients, the imaging information from the lymph nodes themselves should not be overlooked. Almost all studies predicting LNM in NSCLC have ignored the imaging characteristics of lymph node stations, which may be attributed to the issue of harmonizing lymph node regions with the study subjects.

Machine learning (ML) is an emerging computer-based approach that learns from data to identify patterns and outcomes, enabling in-depth examination of interactions between variables and, for certain algorithms, iterative learning to update model parameters (Wu et al., 2020). In terms of feature selection and predictive modeling, ML is currently the most effective tool and is increasingly used to predict lymph node metastasis in NSCLC (Chong et al., 2021; Geng et al., 2022). However, to date, studies have included relatively few patients, and very few have comprehensively integrated imaging, metabolic, and clinical data within a unified analytical framework. This study retrospectively analyzed 1,149 patients, utilizing PET-CT imaging to examine the imaging characteristics, clinical features, and pathological characteristics of resectable NSCLC primary tumors and lymph nodes, aiming to develop an accurate predictive model for MLNM in NSCLC patients.

## Materials and Methods

### Patients

This retrospective cohort included consecutive patients who underwent surgical treatment and were pathologically diagnosed with NSCLC in the First Hospital of Jilin University from January 2017 to December 2023. The dates for accessing data for research purposes were from December 2023 to August 2024. Inclusion criteria were as follows: (1) 18F-FDG PET-CT examination was performed within 2 weeks before surgery; (2) receiving surgical treatment and systematic lymph node dissection; (3) postoperative pathology confirmation of NSCLC. Exclusion criteria were: (1) neoadjuvant therapy; (2) patients with history of other malignant tumors; (3) presence of distant metastasis.

**Figure 1:**
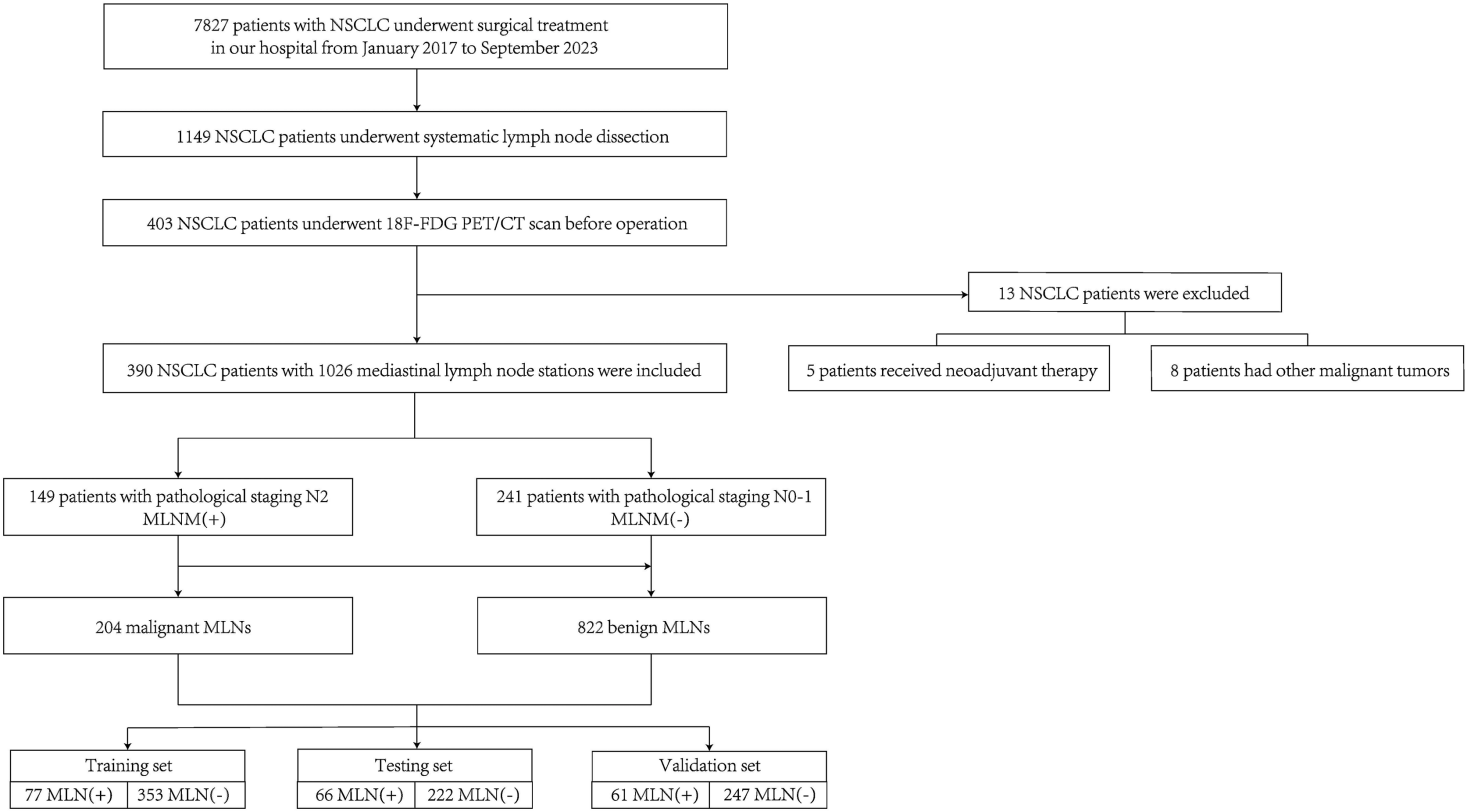
Patient selection flowchart.

Within the study period, a total of 1,149 patients with stage II-III NSCLC who underwent surgical treatment and mediastinal lymph node dissection were identified. Among them, a total of 403 patients underwent 18F-FDG PET-CT examination two weeks before surgery. Five patients had received preoperative adjuvant therapy, and eight patients had a history of other malignant tumors. No patient had distant metastasis. After applying the exclusion criteria, 13 patients were excluded and 390 patients were finally included in the analysis (Fig. 1). The study protocol was reviewed and approved by the Institutional Review Board/Ethics Committee of the first Bethune Hospital of Jilin University (approval No. 2024-1125), which waived the requirement for written informed consent from patients. All methods were carried out in accordance with relevant guidelines and regulations.

### PET-CT scan

All patients underwent the same PET-CT examination. A Biograph 16HR PET-CT scanner (Siemens Biograph 16HR) was used in the study. Before imaging, patients were fasted for more than 6 h, and their blood glucose levels needed to be below 8 mmol/L. 18F-FDG (Sumitomo HM-12, pH 4-8, radioactive purity > 95%, radioactive activity concentration > 370 MBq/ml) was then injected intravenously at a dose of 3.7 MBq/kg. Image acquisition began after 1 h. CT images were scanned from the skull base to the proximal thighs (tube voltage 120 kV, tube current 50 mAs, rotation speed 0.5 s/r, field of view (FOV) 812 mm × 812 mm, 512 × 512 matrix, slice thickness 4 mm). Low-dose CT was used for attenuation correction, and CT images were reconstructed using standard B30f soft tissue. Therefore, positron emission tomography (PET) images were scanned (eight window levels, 2.5 min each, field of view 812 mm × 812 mm, 144 × 144 matrix, slice thickness 4 mm) from the top of the head to the mid-thigh. PET images were reconstructed iteratively in 3D mode using ordered subset expectation maximization (two iterations, 24 subsets, and Gaussian filtering). Both PET and CT images were acquired using a slice thickness of 4 mm during scanning. Subsequently, all images were reconstructed with a slice thickness of 1.25 mm and an increment of 1.0 mm to improve spatial resolution and facilitate volumetric analysis. This process ensured consistent voxel geometry for quantitative feature extraction.

### Image processing

The target lesions in this study were the primary tumor and the dissected mediastinal lymph node stations (stations 2R, 3a, 4R, 4L, 5, 6, 7, 8, 9). On the fused PET-CT images, regions of interest (ROIs) were manually delineated by selecting the cross section with the largest maximum standardized uptake value (SUVmax) centered on the lesion and lymph node region. PET images were reconstructed iteratively in 3D mode to generate volumes of interest (VOIs). SUVmax ≥ 40% was used as the standardized uptake value (SUV) threshold to determine the final contour edge of the target. The imaging features were recorded. The imaging features were recorded after ROIs were delineated on fused PET-CT images. Quantitative parameters, including SUVmax, mean standardized uptake value (SUVmean), peak Standardized Uptake Value (SUVpeak), maximum Standardized Uptake Value normalized to Lean Body Mass (SULmax), mean Standardized Uptake Value normalized to Lean Body Mass (SULmean), MTV, and TLG, were automatically extracted using the Medex imaging system, while qualitative CT features (*e.g*., spiculation, lobulation, bronchial cutoff, pleural indentation, and calcification) were assessed visually by two experienced nuclear medicine physicians. VOIs were reviewed and corrected by two nuclear medicine physicians, each with approximately 15 years of independent clinical experience, who were blinded to pathological and clinical information, and any discrepancies were resolved by consultation. The dissected mediastinal lymph node area was measured according to the lymph node map of coronal, sagittal and transverse CT published by the International Association for the Study of Lung Cancer (IASLC) in 2009.

### Pathological diagnosis

All patients underwent lobectomy or partial pneumonectomy with systematic hilar (N1) and mediastinal lymph node (N2) dissection within 2 weeks after 18F-FDG PET-CT examination. The pathological status of mediastinal lymph nodes involved in this study was determined by postoperative pathological N (pN) staging according to the postoperative pathological results. Pathological findings were determined by two senior pathologists, each with approximately 15 years of professional diagnostic experience, and any disagreements were resolved by consultation.

### Feature extraction

After data preprocessing, feature selection was conducted as follows. Pathological features: The pathological status (metastatic/non-metastatic) of each station of resected mediastinal lymph nodes, histological type of primary tumor, microhistological type, maximum diameter of tumor, necrosis, nerve, blood vessel, visceral pleural invasion or dissemination along the airway, TNM staging were extracted from the pathological information system of the Department of Pathology of the First Hospital of Jilin University. TNM staging was determined according to the eighth edition of the staging system.

Imaging features: Medex imaging system was used to extract the location of lung cancer (divided into central type and peripheral type according to the inner 1/3 and outer 2/3 of the lung field), the location of tumor, the type of lung nodule (pure ground glass nodule, mixed density and implementation nodule). There were spiculation sign, lobulation sign, bubble sign, cavity, calcification, bronchial truncation sign, pleural indentation sign and pleural effusion. Whether the boundary of the lesion was clear and whether it was complicated with other pulmonary diseases (Chronic Obstructive Pulmonary Disease (COPD), pneumonia, bronchiectasis, obstructive atelectasis). Imaging parameters included the maximum CT attenuation value of the primary lesion and each resected mediastinal lymph node station (CTmax, expressed in Hounsfield units, HU), mean CT attenuation value (CTavg, HU), SUVmax, Minimum Standardized Uptake Value (SUVmin), average standardized uptake value (SUVavg), SUVpeak, Standard Deviation of Standardized Uptake Value (SUVsd), SULmax, Minimum Standardized Uptake Value normalized to Lean Body Mass (SULmin), Average Standardized Uptake Value normalized to Lean Body Mass (SULavg), Peak Standardized Uptake Value normalized to Lean Body Mass (SULpeak), Standard Deviation of Standardized Uptake Value normalized to Lean Body Mass (SULsd), MTV, and TLG. The clinical stage (cN) of the lymph node and the short diameter S(mm) of the lymph node.

Clinical features: Gender, age, smoking, drinking, hypertension, type 2 diabetes, and coronary heart disease were extracted from the HALO clinical record system of the First Hospital of Jilin University. Clinical laboratory indicators included D-dimer (mg/L) and erythrocyte sedimentation rate (mm/1 h). Tumor markers included cytokeratin 19 fragment (Cyfra21-1, ng/mL), ProGRP (pg/mL), CA724 (U/mL), CEA (ng/mL), CA242 (U/mL), CA125 (U/mL), neuron-specific enolase (NSE, ng/mL), AFP (ng/mL), SCC-Ag (ng/mL), CA19-9 (U/mL), and CA153 (U/mL). Pulmonary function was represented by FEV_1_ (L).

### Statistical analysis

All data from January 2017 to December 2023 were analyzed using SPSS (version 27; IBM, Armonk, NY, USA) and performed the analysis. Statistical analyses were performed using SPSS version 27 (IBM Corp., Armonk, NY, USA). Continuous variables were tested for normality. Normally distributed data were expressed as mean ± SD and compared using the t-test, while non-normal data were shown as median (IQR) and compared using the Mann–Whitney U test. Categorical variables were compared using the $\chi^2$ test or Fisher’s exact test, and their utilization rates were calculated accordingly. Correlations between variables were analyzed using Spearman’s rank correlation. A two-sided *P* < 0.05 was considered statistically significant. Some patients in this retrospective study did not have complete data available for all clinical or imaging variables. To minimize bias, variables with less than 10% missingness were imputed using the median (for continuous variables) or mode (for categorical variables). Variables with more than 20% missing data were excluded from model development. A multiple imputation approach (five imputations using chained equations) was performed as a sensitivity analysis to confirm the robustness of the results. Cases with missing dependent variables were excluded from model training and evaluation.

### Feature selection

Random forest based Out-of-Bag (OOB) error estimation method was used to select clinical features, pathological features and imaging features. The Bootstrap sampling technique was used to construct the training set by extracting samples from the original dataset, while the unselected samples were used as out-of-pocket data to evaluate the generalization performance of the model. By analyzing the out-of-pocket error changes before and after each feature was added to the model, we were able to quantify the impact of each feature on the prediction performance of the model and thus identify key features. Shapley Additive Explanations (SHAP) values were introduced to account for the contribution of features to the model output. The SHAP method assigns each feature a contribution score based on game theory, which helps us to understand the importance of the feature and its relationship with the target variable. A 90% cumulative contribution threshold was set, and the top n most influential features were selected for inclusion in the machine-learning algorithm. In total, 41 imaging features were initially extracted from both primary tumors and mediastinal lymph nodes. To prevent model overfitting and improve interpretability, features were ranked by their mean decrease in the OOB error and SHAP importance values. Features contributing cumulatively to 90% of the total model variance were retained, resulting in the selection of 20 imaging features. This threshold was empirically determined to balance model simplicity and predictive accuracy. The retained features included metabolic indices (*e.g*., SUVmax, MTV, TLG), anatomical measurements (*e.g*., lymph node short-axis diameter, CTmax, CTavg), and qualitative CT signs (*e.g*., spiculation and pleural indentation). These features were subsequently combined with the top-ranked clinical and pathological variables identified through the same selection process to form the final input set for model construction. SHAP analysis was performed separately for clinical, pathological, and imaging feature sets to interpret domain-specific feature contributions. In addition, a combined SHAP analysis was conducted for the integrated TLPC model to visualize the overall feature importance across domains.

All machine-learning analyses were implemented in Python 3.8 (Python Software Foundation) within a reproducible computing environment. The core dependent libraries and their versions were as follows: numpy 1.24.3, pandas 2.1.0, scikit-learn 1.3.0, xgboost 1.7.5, shap 0.43.0, and matplotlib 3.8.0.

A fixed random seed (2024) was applied throughout data processing, feature selection, and model training to ensure consistent results across runs. All model training, validation, and visualization procedures were executed using these fixed package versions to enable full reproducibility of the analytical pipeline.

### Select the optimal machine learning classifier

Supervised learning algorithms were implemented to construct and evaluate predictive models. The performance of various machine learning algorithms was further evaluated in order to identify the optimal predictive model. The explored algorithms include Logistic Regression, Classification and Regression Tree (CART), SVM, gradient boosting decision tree (GBDT), Random Forest, multi-layer perceptron (MLP), extreme gradient boosting tree (XGBoost) and k-nearest neighbor algorithm (KNN). To ensure the robustness of the model, the dataset was divided into a training set (*n* = 410), a test set (*n* = 308), and a validation set (*n* = 308) using a stratified random sampling strategy based on the target variable (presence or absence of MLNM). This ensured that the proportion of positive and negative cases was consistent across all subsets, making each set representative of the overall dataset and preventing sampling bias. During the training process, each algorithm was subjected to k-fold cross-validation to assess its stability and generalization performance through repeated partitioning of the training and validation sets. Cross-validation provides reliable performance estimates for each model, avoiding the risk of model overfitting or underfitting. In addition, the area under the receiver operating characteristic (ROC) curve (AUC) was employed as the primary metric for model evaluation. The AUC value can not only comprehensively measure the performance of classifiers, but also provide an objective basis for the comparison between different algorithms. By comparing the AUC values of each model, the algorithm with the best performance and strongest stability was selected as the core model for predicting MLNM.

### Model tuning and hyperparameter optimization

To ensure fair and reproducible model comparison, all machine-learning classifiers were trained and tuned using standardized pipelines implemented in Python 3.8 (scikit-learn 1.3.0, xgboost 1.7.5). The full cohort was first randomly split into a training set, a held-out internal test set, and an external validation set (as described in the ‘Statistical analysis’ section). Within the training set, a nested cross-validation framework was employed to optimize hyperparameters and avoid overfitting or information leakage. Specifically, the training set was internally divided into five folds: an inner loop for hyperparameter optimization using Randomized Search CV, and an outer loop for unbiased model evaluation.

Each algorithm was tuned within a literature-based search space as follows: Logistic Regression: penalty (L1/L2), C (10^−4^–10^2^), solver (liblinear/saga); CART: max_depth (2–20), min_samples_split (2–20), criterion (gini/entropy); Random Forest: n_estimators (200–1,500), max_depth (5–30 or None), max_features (sqrt/log2), bootstrap (True/False); GBDT/XGBoost: n_estimators (100–1,000), learning_rate (0.01–0.3), max_depth (3–10), subsample (0.6–1.0), colsample_bytree (0.6–1.0), min_child_weight (1–10), gamma (0–5); SVM with radial basis function kernel: C (10^−3^–10^3^), γ (10^−4^–10^0^); KNN: n_neighbors (3–51, step 2), weights (uniform/distance); MLP: hidden_layer_sizes ∈ {(64,), (128,), (64,32)}, activation (relu/tanh), learning_rate_init (10^−4^–10^−3^), max_iter (500), early_stopping (True).

The optimal hyperparameter combination for each algorithm was selected according to the mean AUC across the inner folds. After tuning, each model was retrained on the full training set with the best parameters and then evaluated on the held-out internal test and independent validation sets. Model probabilities were calibrated using Platt scaling or isotonic regression according to the lowest cross-validated Brier score.

For reproducibility, a fixed random seed (2024) was applied to all procedures. Core software packages included numpy 1.24.3, pandas 2.1.0, matplotlib 3.8.0, and shap 0.43.0.

### Development and validation of the prediction model

To predict MLNM, three prediction models including TLPC, Tumor-Pathology-Clinical (TPC) and Lymph-Pathology-Clinical (LPC) were established in the test set according to the selected clinical factors, tumor PET-CT imaging features, lymphatic PET-CT imaging features and the combination of the above features. The three prediction models were further validated in the validation set. Decision curve analysis (DCA) was used to evaluate the clinical utility of a model by measuring the net benefits at different thresholds. To compare the clinical utility of eight machine learning algorithms in establishing prediction models. Accuracy, precision, recall and F1-score were used to quantify the diagnostic effect of the prediction model. The traditional standard (SUVmax > 2.5 or S > 1 cm) prediction model was used for comparison. The AUC was used to quantify the diagnostic performance of the prediction model, and the DeLong test was used to evaluate the difference in AUC between each model. All *P*-values were derived from DeLong’s test comparing AUCs between models, with statistical significance defined as *P* < 0.05. Given that multiple models and feature sets were compared (eight classifiers across three feature combinations), a correction for multiple comparisons was applied to control the family-wise error rat. The *P*-values obtained from DeLong’s tests were adjusted using the Benjamini–Hochberg false discovery rate (FDR) method, with an adjusted significance threshold of q < 0.05. This approach reduces the risk of type I error inflation while maintaining statistical power in comparative model evaluation. The confusion matrix was used to visually present the classification effect of the model, including statistics for true positive, false positive, true negative, and false negative.

## Results

### Characteristics of the patients

This study included a total of 390 patients with NSCLC, among whom 241 patients (61.79%) were classified as pN0-1, while 149 patients (38.21%) were classified as pN2 (Table 1). A total of 1,026 mediastinal lymph node stations were dissected, with 204 stations (19.88%) showing metastasis and 822 stations (80.12%) exhibiting no metastasis. The clinical, pathological, and imaging characteristics were analyzed and evaluated in the training set (*n* = 433), test set (*n* = 288), and validation set (*n* = 308). To improve methodological clarity and readability, Table 1 was divided into two parts: one summarizing clinical and pathological characteristics, and the other summarizing imaging and radiomic features. Continuous variables were rounded to one decimal place, and all abbreviations were defined in table footnotes for consistency and transparency.

**Table 1:** Baseline characteristics and imaging features.

Variable	Overall (*n* = 390)	pN0–1 (*n* = 241)	pN2 (*n* = 149)	*P*-value
Age (years)	63.2 ± 8.5	62.7 ± 8.3	64.1 ± 8.7	0.214
Sex, *n* (%)	228/162	138/103	90/59	0.371
Smoking history, *n* (%)	210 (53.8)	118 (49.0)	92 (61.7)	0.021
Hypertension, *n* (%)	115 (29.5)	70 (29.0)	45 (30.2)	0.812
Diabetes, *n* (%)	78 (20.0)	44 (18.3)	34 (22.8)	0.301
Histological subtype, *n* (%)	–	–	–	0.041
Adenocarcinoma	276 (70.8)	181 (75.1)	95 (63.8)	
Squamous cell carcinoma	95 (24.4)	49 (20.3)	46 (30.9)	
CEA (ng/mL)	3.2 [1.8–5.9]	2.9 [1.6–4.7]	4.8 [2.2–7.5]	0.008
Cyfra21-1 (ng/mL)	3.1 [2.0–5.3]	2.7 [1.9–4.8]	4.2 [2.5–6.3]	0.011
SCC-Ag (ng/mL)	1.1 [0.7–2.0]	1.0 [0.6–1.8]	1.3 [0.8–2.4]	0.042
TNM stage (8th edition)	–	–	–	<0.001
Stage I–II	237 (60.8)	184 (76.3)	53 (35.6)	
Stage III	153 (39.2)	57 (23.7)	96 (64.4)	

Feature	pN0–1 (*n* = 241)	pN2 (*n* = 149)	*P*-value
Tumor location (central/peripheral)	72/169	83/66	<0.001
SUVmax (tumor)	8.7 ± 3.9	12.1 ± 4.5	<0.001
SUVavg (tumor)	5.6 ± 2.7	7.9 ± 3.2	<0.001
MTV (cm^3^)	6.5 [3.1–13.8]	11.2 [6.2–24.9]	0.002
TLG (g)	32.4 [18.7–58.9]	61.7 [35.1–97.3]	<0.001
CTmax (HU)	78.3 ± 22.1	91.5 ± 27.8	0.015
Lobulation sign, *n* (%)	148 (61.4)	112 (75.2)	0.013
Spiculation sign, *n* (%)	95 (39.4)	81 (54.4)	0.011
Bronchial cutoff sign, *n* (%)	54 (22.4)	48 (32.2)	0.046
Pleural indentation sign, *n* (%)	86 (35.7)	65 (43.6)	0.128
Lymph node short-axis diameter (mm)	8.9 ± 2.1	13.7 ± 3.4	<0.001
Lymph node SUVmax	2.1 ± 0.8	4.8 ± 1.7	<0.001

**Note:**

CEA, Carcinoembryonic antigen; Cyfra21-1, cytokeratin 19 fragment; SCC-Ag, squamous cell carcinoma antigen; TNM, tumor-node-metastasis; pN, pathologic nodal stage; SUVmax, maximum standardized uptake value; SUVavg, mean standardized uptake value; MTV, metabolic tumor volume; TLG, total lesion glycolysis; CTmax, maximum CT attenuation; HU, Hounsfield units.

**Figure 2:**
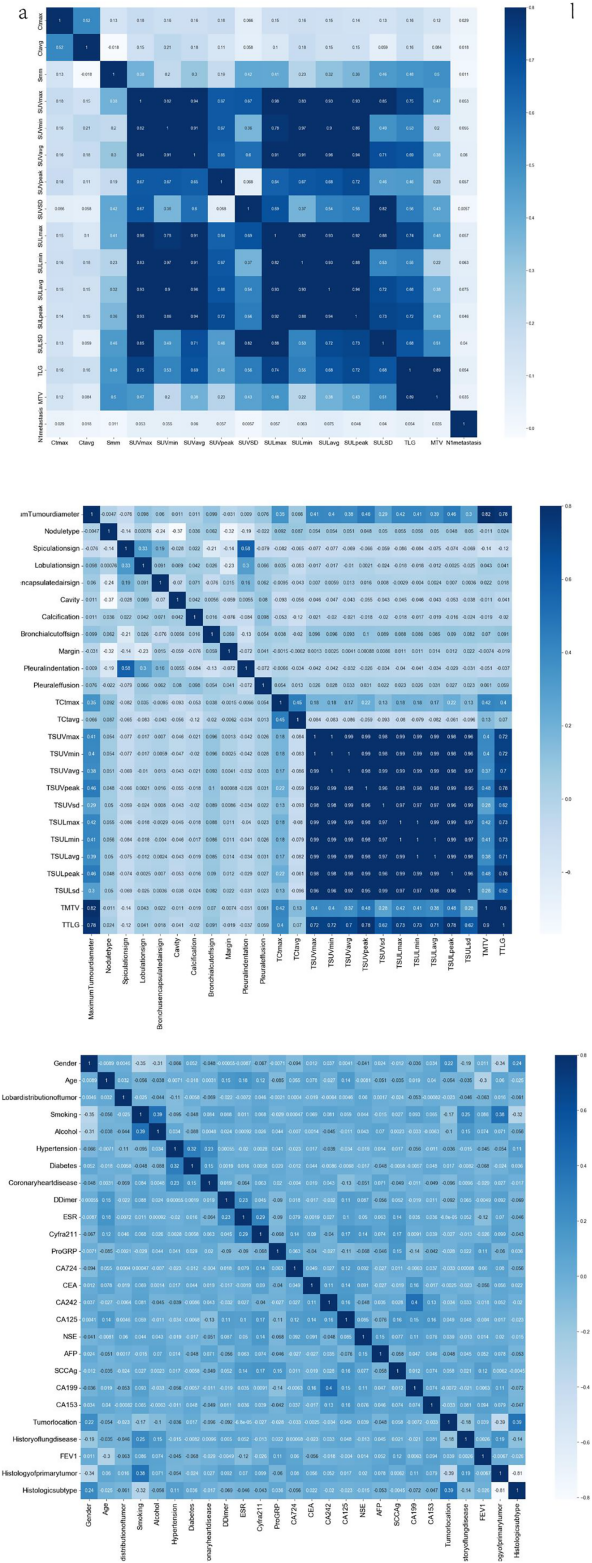
Heat map of Spearman correlation coefficients of clinicopathological and imaging features. (A) Correlation matrix of PET-CT imaging quantitative features, including SUV, SUL, MTV, and TLG parameters. (B) Correlation matrix of radiological morphological characteristics (*e.g*., spiculation, lobulation, bronchus sign) and PET-CT parameters. (C) Correlation matrix of clinical and serological indicators, including demographic data, comorbidities, and tumor markers (CEA, CA19-9, *etc*.). Color scale represents Pearson correlation coefficients (r). Darker blue indicates stronger positive correlation.

Figure 2 presents a heatmap of the Spearman correlation analysis, illustrating the relationships among tumor and lymph node metabolic characteristics, imaging features, clinical-pathological characteristics, and various clinical factors. Figure 2A shows strong correlations among metabolic parameters (SUVmax, SUVavg, MTV, and TLG) with Spearman coefficients ranging from 0.71 to 0.96. Figure 2B demonstrates moderate correlations between lymph node and primary tumor imaging features (0.1–0.6). Figure 2C depicts associations among clinical variables such as smoking history, forced expiratory volume in 1 s (FEV1), CEA, and SCC-Ag, revealing an inverse relationship between smoking and FEV1 and weak correlations between tumor markers and traditional clinical indicators.

Cross-domain correlations showed that several metabolic parameters (SUVmax, MTV, TLG) were moderately associated with systemic clinical markers (*e.g*., CEA, Cyfra21-1), suggesting biologically plausible links between tumor metabolism and systemic inflammation. To minimize the impact of potential multi-collinearity, tree-based models (XGBoost, Random Forest) intrinsically handled redundant predictors through random feature sampling, and additional variance inflation factor (VIF) screening confirmed that no variables exceeded VIF > 5. These correlations guided subsequent feature selection and informed the integration of multimodal variables in model construction.

### Results of feature selection

**Figure 3:**
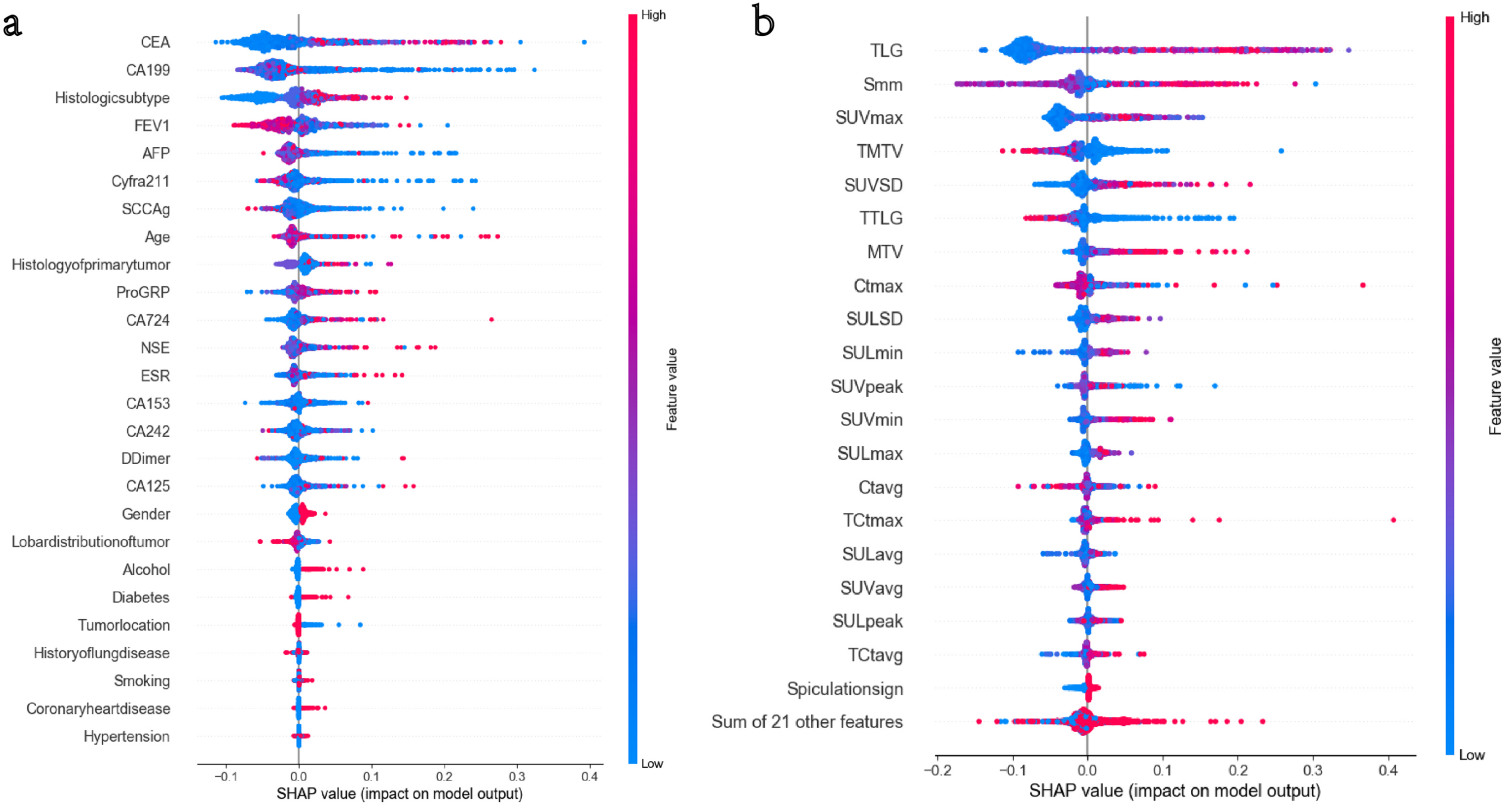
(A and B) The top 15 clinical and pathological variables in terms of feature importance. The top 20 imaging variables in terms of feature importance.

This study extracted 41 imaging features from primary tumors and mediastinal lymph nodes, selecting the top 20 features based on their contribution for the machine learning model. These key features include CT values of lymph nodes (maximum and average), TLG, MTV, and SUV metabolic parameters (such as SUVmax and SUVpeak). Additionally, anatomical features such as spiculated signs and lymph node short diameter (Smm) were incorporated, as they significantly influence MLNM prediction. From the clinical and pathological characteristics, 26 indicators were extracted, ultimately identifying 15 important features, including CEA, CA19-9, tumor pathology type, FEV1, AFP, and Cyfra211. These features exhibited high relevance in MLNM prediction. SHAP analysis in Fig. 3A indicates that metabolic features such as TLG, SUVmax, and MTV contribute the most to the model, highlighting that metabolic activity and tumor burden are crucial predictors of MLNM. The short diameter of lymph nodes (Smm), as an anatomical feature, also shows significant contribution, further supporting its value in morphological prediction. Figure 3B demonstrates that CEA, CA19-9, and tumor histological type are the primary clinical and pathological driving factors, with FEV1, as a lung function indicator, also impacting the model’s results. The independence and importance of these features are clearly defined through SHAP values, providing direction for future model optimization and clinical application.

### Performance of machine learning algorithms

**Figure 4:**
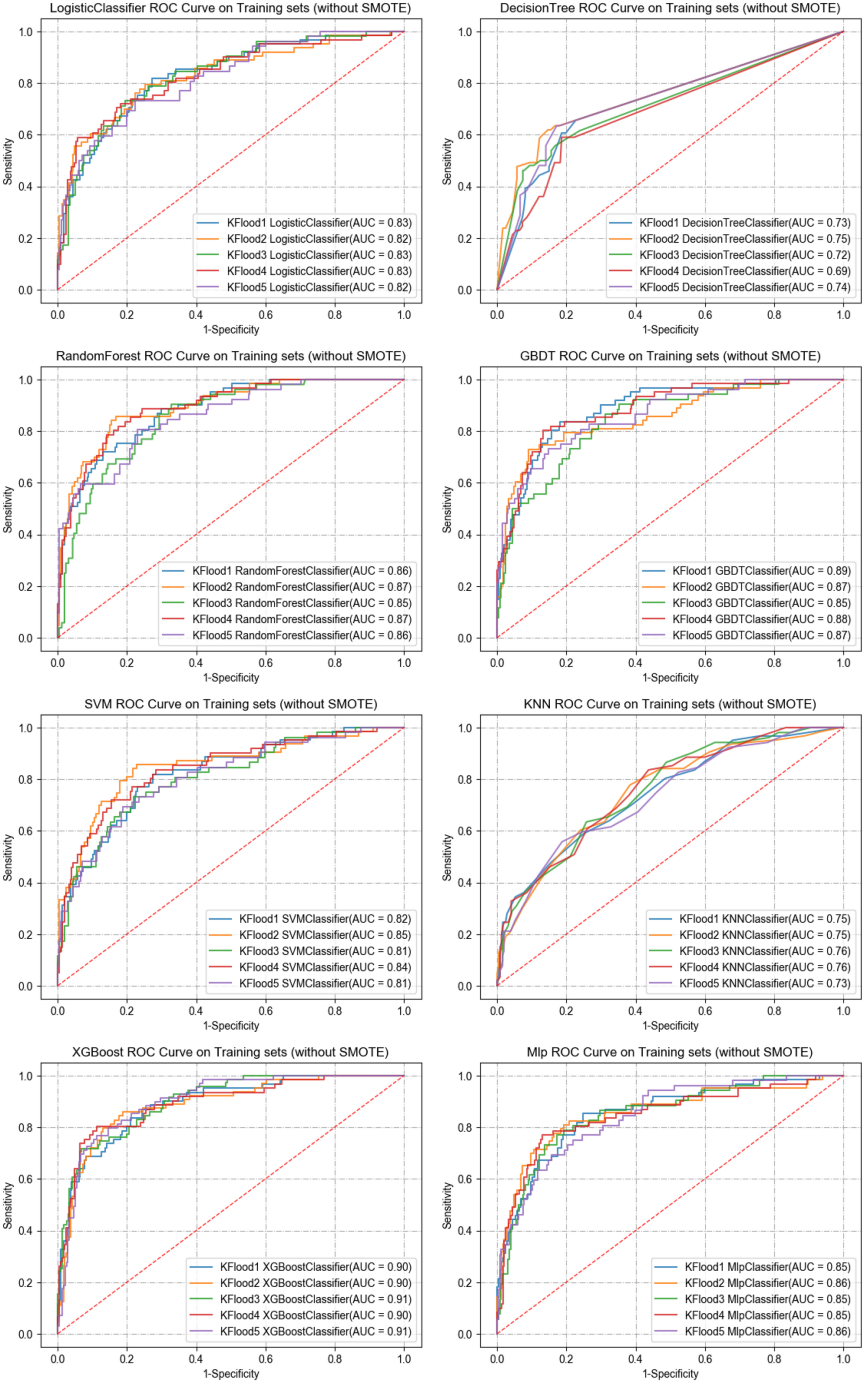
ROC curves of the eight ML algorithms for the training set.

**Figure 5:**
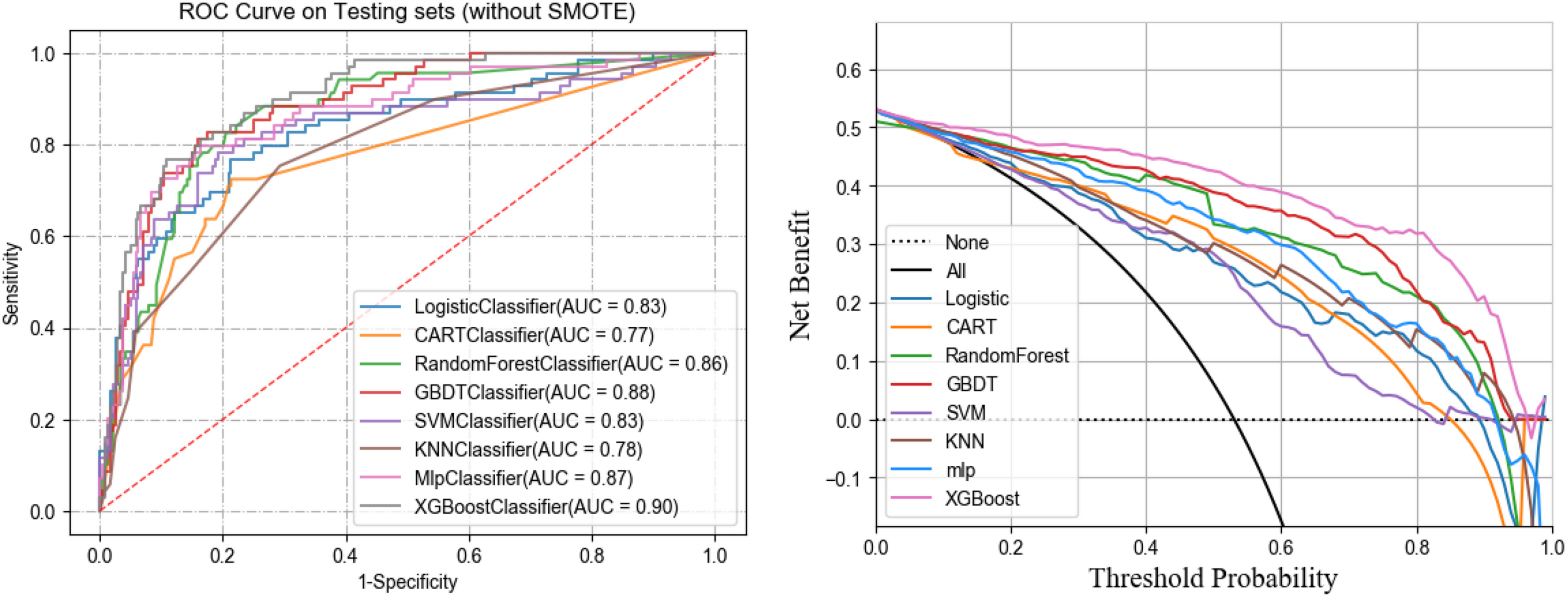
ROC and DCA curves of eight ML models on the testing set.

In the training set, five-fold cross-validation was used to evaluate the performance of various machine learning algorithms. The results indicated that XGBoost performed the best, achieving an AUC of 0.90. In the test set, the AUCs for each algorithm were as follows: logistic regression (0.83), SVM (0.83), GBDT (0.88), Random Forest (0.86), CART (0.77), MLP (0.87), and KNN (0.78). Notably, XGBoost had the highest AUC in both cross-validation and test sets at 0.90, significantly outperforming other models (see Table 2). The clinical decision curve further validated the advantages of XGBoost. Within the threshold range of 0 to 1, XGBoost demonstrated high net benefits across all possible thresholds, clearly surpassing other algorithms, indicating the model’s potential for clinical application. Figures 4 and 5 present the ROC curves and clinical decision curves for each machine learning algorithm in the training and validation sets. XGBoost excelled across multiple evaluation metrics, including AUC, accuracy, recall, and F1-score, confirming its stability and superiority in predicting MLNM. Therefore, based on these evaluation results, XGBoost was selected as the optimal classifier for MLNM prediction, and its use is recommended in future clinical applications and further research.

**Table 2:** Model performance across machine learning algorithms.

Algorithm	Training AUC	Test AUC	Validation AUC	Accuracy (%)
Logistic regression	0.84	0.83	0.80	81.2
SVM	0.85	0.83	0.82	82.0
Random Forest	0.89	0.86	0.84	84.5
GBDT	0.90	0.88	0.85	85.3
CART	0.78	0.77	0.74	75.1
MLP	0.88	0.87	0.84	83.7
KNN	0.80	0.78	0.76	77.0
XGBoost	0.91	0.90	0.87	87.2

**Note:**

SVM, Support vector machine; GBDT, gradient boosting decision tree; CART, classification and regression tree; MLP, multilayer perceptron; KNN, k-nearest neighbors; AUC, area under the receiver operating characteristic curve.

**Figure 6:**
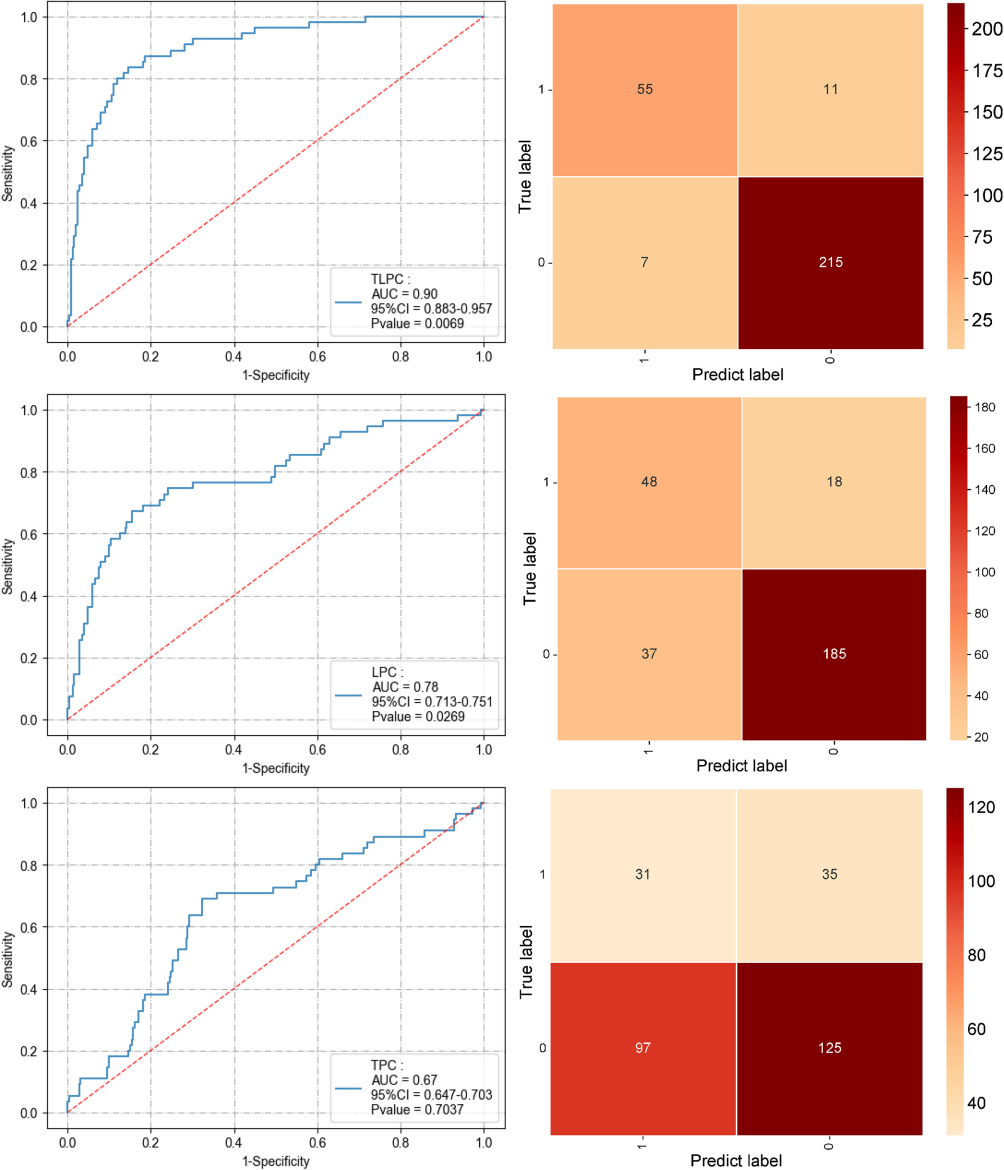
Performance of the three prediction models after applying the XGBoost algorithm to the test set (ROC curve and confusion matrix).

To comprehensively compare the effectiveness of the TPC, LPC, and TLPC models in predicting MLNM, evaluations were first conducted on the test set (see Fig. 6 and Table 3). The results indicated that the integrated TLPC model achieved the highest performance, with an AUC of 0.90 (95% CI [0.883–0.957]), specificity of 0.84, and sensitivity of 0.96 (*P* = 0.0069). In contrast, the LPC model achieved an AUC of 0.78 (95% CI [0.713–0.751]), with a specificity of 0.73 and sensitivity of 0.84 (*P* = 0.0269). The TPC model had an AUC of only 0.67 (95% CI [0.647–0.703]), with a specificity of 0.46 and sensitivity of 0.56 (*P* = 0.7037), demonstrating significantly inferior performance.

**Table 3:** Comparative diagnostic performance of three prediction models for mediastinal lymph node metastasis.

Model	AUC (95% CI)	Sensitivity	Specificity	*P*-value
TLPC	0.90 [0.883–0.957]	0.96	0.84	0.0069
LPC	0.78 [0.713–0.751]	0.84	0.73	0.0269
TPC	0.67 [0.647–0.703]	0.56	0.46	0.7037
Traditional (SUVmax > 2.5 or S > 1 cm)	0.71 [0.673–0.732]	0.74	0.70	—

**Note:**

TPC, Tumor-Pathology-Clinical model; LPC, Lymph-Pathology-Clinical model; TLPC, Tumor-Lymph-Pathology-Clinical model; SUVmax, maximum standardized uptake value; S, lymph node short-axis diameter; AUC, area under the receiver operating characteristic curve; CI, confidence interval.

**Figure 7:**
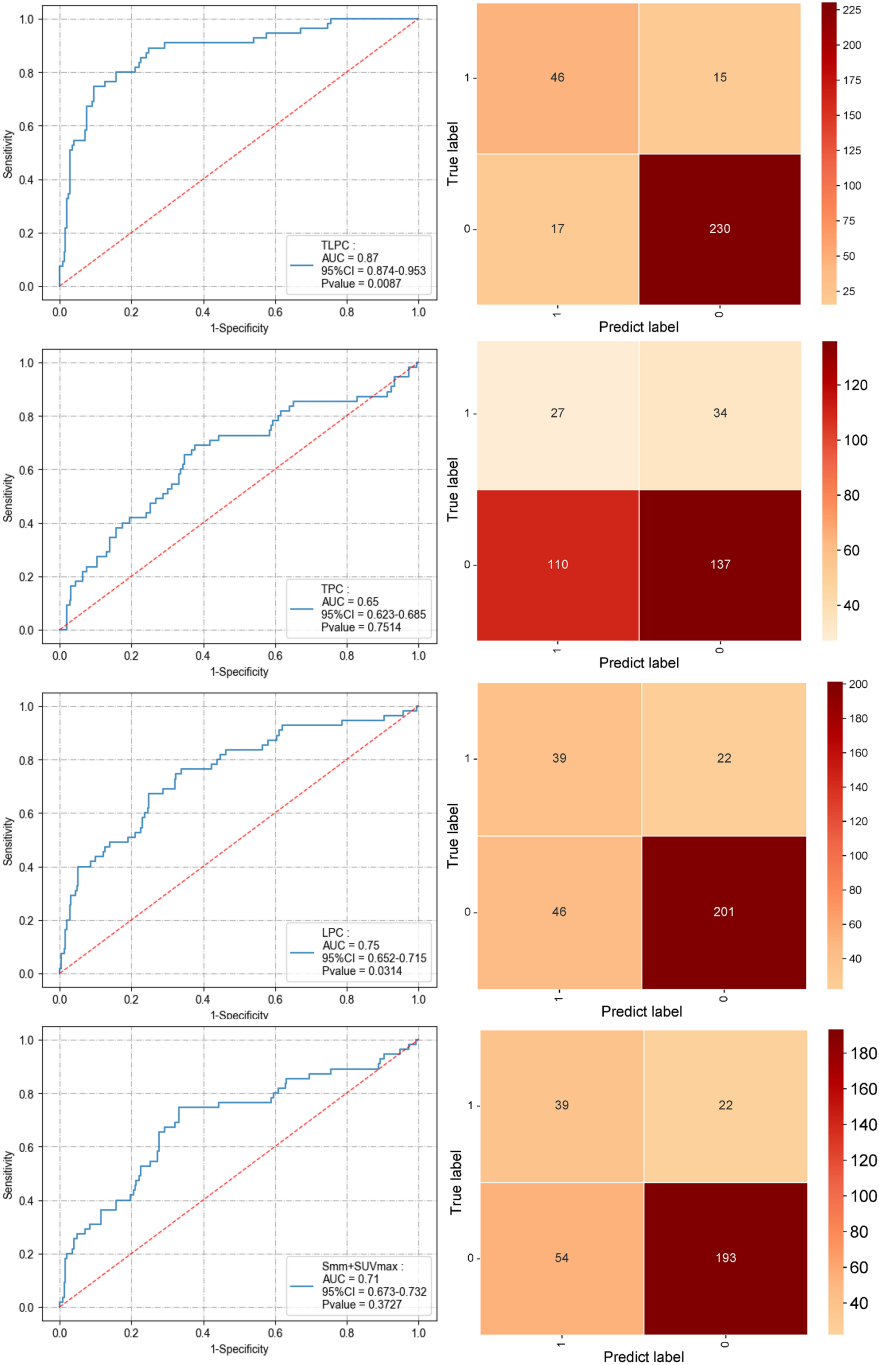
Performance of the three prediction models after applying the XGBoost algorithm to the validation set and the performance of the S+SUVmax method (ROC curve and confusion matrix).

In the validation set, the TLPC model similarly exhibited superior performance, with an AUC of 0.87 (95% CI [0.874–0.953]), specificity of 0.76, and sensitivity of 0.93 (*P* = 0.0087), again outperforming the other models. The LPC model recorded an AUC of 0.68 (95% CI [0.652–0.715]), with a specificity of 0.64 and sensitivity of 0.78 (*P* = 0.0314). The TPC model had an AUC of 0.65 (95% CI [0.623–0.685]), specificity of 0.44, and sensitivity of 0.55 (*P* = 0.7514), which was still suboptimal. Compared to traditional diagnostic methods (AUC of 0.71, 95% CI [0.673–0.732], specificity of 0.70, sensitivity of 0.74), the TLPC model demonstrated a significant advantage in predicting MLNM (Fig. 7 and Table 3). The TLPC model not only dominated in AUC values but also showed higher specificity and sensitivity, confirming its potential for clinical application. This indicates that the integrated model can predict MLNM more comprehensively and accurately. It highlights that combining metabolic and anatomical features of the primary tumor and lymph nodes, along with pathological and clinical characteristics, significantly enhances the predictive performance of the model.

## Discussion

The correlation analysis revealed meaningful interrelationships across imaging, metabolic, and clinical domains. Specifically, smoking history showed a negative correlation with FEV1, while metabolic indicators such as SUVmax, MTV, and TLG demonstrated moderate associations with tumor markers including CEA and Cyfra21-1, reflecting potential biological links between tumor metabolism and systemic inflammation. These findings suggest that clinical, metabolic, and anatomical imaging features provide distinct yet complementary information, thereby justifying the multimodal integration strategy adopted in this study.

This study developed three different combination models (TPC, LPC, and TLPC) to validate the significance of imaging, pathological, and clinical features in predicting MLNM in patients with NSCLC. The strong internal correlations among SUV-based parameters (*e.g*., SUVmax, SUVavg, MTV, and TLG) indicate a certain degree of redundancy within metabolic variables, supporting the dimensionality reduction applied during feature selection. Conversely, the weak associations between metabolic and anatomical CT features suggest that integrating both modalities enhance the representational richness of the predictive model. Additionally, the observed weak correlations between tumor markers and demographic or physiological variables support the notion that clinical indicators contribute unique, non-overlapping information for model construction.

Together, these results justify the multimodal integration strategy adopted in the TLPC model, which demonstrated the best predictive performance by leveraging complementary data from imaging, pathology, and clinical domains. The TLPC model performed best, with an AUC of 0.90, significantly outperforming the other models. This underscores the critical importance of multimodal data integration in enhancing the predictive accuracy of MLNM. Compared with traditional single-feature models, the TLPC model excelled in both sensitivity and specificity, particularly with the XGBoost algorithm, which demonstrated advantages in handling multidimensional data and optimizing predictive models. This provides an effective tool for precise preoperative staging and personalized treatment.

The successful establishment of the model in this study holds significant implications for the staging of NSCLC, as accurate staging is crucial for determining treatment strategies. In particular, evaluating mediastinal lymph node involvement is essential. According to the latest clinical guidelines, patients classified as N0 or N1 are considered suitable candidates for surgical intervention, while those with N2 disease typically require neoadjuvant therapy prior to surgery (Bradley et al., 2025; Ettinger et al., 2023; Remon, Soria & Peters, 2021). Although imaging examinations such as PET-CT are commonly used for lymph node assessment, their sensitivity and specificity remain limited, with recent real-world series reporting sensitivities around 0.80–0.85 and specificities around 0.85–0.90 for N-stage assessment (Khan et al., 2025; Nakanishi et al., 2021). A recent head-to-head meta-analysis reported pooled sensitivity and specificity of 0.82 and 0.88 for 18F-FDG PET-CT in detecting nodal metastases, comparable to PET-MRI (Yu & Chen, 2024). Furthermore, while EBUS-TBNA can aid in diagnosing mediastinal lymph node metastasis, it is an invasive procedure with a reported false-negative rate ranging from 15% to 25% in clinical studies (Eapen et al., 2013; Huang et al., 2024; Matache et al., 2025; Wang et al., 2024). Several recent studies have proposed PET-CT-based ML models for mediastinal staging. For example, Rogasch et al. (2023) developed a gradient-boosting classifier using routinely available PET-CT and clinical variables, achieving an AUC of 0.91 for differentiating N0/1 from N2/3 disease.

In the study, the ability of PET-CT to predict MLNM positivity in NSCLC patients was first assessed (AUC = 0.71). Subsequently, a new model was constructed by combining the radiological features of the primary tumor and lymph nodes with clinical characteristics. This model achieved an AUC value of up to 0.90 (sensitivity of 0.90, specificity of 0.84), significantly outperforming models that relied solely on imaging or pathological features. Our model achieved excellent predictive efficiency for N2 staging in NSCLC, suggesting that combining radiological and clinical characteristics of the primary tumor and lymph nodes can significantly enhance the predictive efficiency for MLNM in NSCLC patients. Similarly, Wang et al. (2023) also demonstrated that the TLPC model outperformed the TPC and LPC models in predicting LNM in NSCLC. However, their study primarily focused on radiomics, with limited inclusion of clinical indicators and a smaller number of patients and lymph nodes. Although the predictive capability of radiomics studies is relatively high (AUC = 0.82–0.88), the lack of clinical features limits the clinical applicability of these models (Wang et al., 2023). In recent years, preoperative lymph node metastasis prediction models incorporating medical imaging have gained significant attention (Li et al., 2025). For example, a positive standard of LN-SUVmax > 2.5 from PET-CT showed a total sensitivity and specificity of 81.3% and 79.4%, respectively (Wang et al., 2024). However, variations in datasets and methods used across different studies have led to discrepancies in the predictive performance for lymph node metastasis.

A key strength and novelty of the present study is the development of an integrated TLPC model that simultaneously incorporates volumetric 18F-FDG PET-CT features from both the primary tumor and mediastinal lymph node stations together with detailed clinicopathologic variables. In contrast to most previous models that relied solely on tumor-based radiologic characteristics or limited clinical factors, our approach explicitly models each lymph node station as a separate prediction unit using station-level annotations, thereby providing a more fine-grained assessment of mediastinal involvement. Moreover, the relatively large, single-center cohort with systematic mediastinal lymph node dissection and rigorous station-level labeling reduces misclassification bias and enhances the internal validity of the model. Compared with the TPC and LPC models, the TLPC model leverages complementary information from both primary tumors and regional lymph nodes and therefore achieves superior discrimination for MLNM.

These findings have several potential clinical implications. First, a reliable station-level prediction of mediastinal lymph node metastasis may help refine preoperative N2 risk stratification and guide decisions regarding invasive mediastinal staging procedures and neoadjuvant therapy in patients with stage II–III NSCLC. Second, many of the PET-CT and clinicopathologic variables used in the TLPC model are routinely collected in contemporary clinical practice, which facilitates implementation and meaningful replication in other institutions. Future work will focus on external validation in multi-center cohorts and prospective evaluation of whether TLPC-guided decision-making can improve patient selection and clinical outcomes.

This study advanced beyond conventional radiomics by incorporating multimodal data—clinical, pathological, and imaging characteristics—to better reflect the complexity of real-world clinical decision-making. Previous research has indicated a significant correlation between patients’ clinical and pathological features and lymph node metastasis. For example, Liao et al. (2023) found that age over 60, smoking history, CEA levels, and the SUVmax of tumors were independent predictive factors for lymph node metastasis in NSCLC patients. Additionally, a significant association between serum D-dimer levels and MLNM in NSCLC was identified in our previous research (Gao et al., 2018). In this study, a 3D volumetric measurement approach was employed to assess and document the radiological characteristics of each resected mediastinal lymph node. MLNM prediction at the station level was achieved by correlating lymph node stations with pathological results. Models that combine tumor and MLNM characteristics demonstrated better predictive performance than those combining clinical and pathological features of the tumor or MLNM alone (Botta et al., 2020; Cong et al., 2020). To our knowledge, this study is the first to integrate imaging features of both tumors and mediastinal lymph nodes with clinical pathological features and apply machine learning techniques to establish multiple predictive models for MLNM in NSCLC patients.

Machine learning, particularly XGBoost, demonstrates significant advantages in predicting MLNM. In our study, the XGBoost model achieved an AUC of 0.90, significantly outperforming other machine learning classifiers such as random forests, SVM, and KNN (Li et al., 2022; Yuan et al., 2022). XGBoost combines gradient boosting with regression trees, effectively capturing complex relationships between nonlinear features, which is crucial for integrating multidimensional data (such as imaging, clinical, and pathological characteristics) (Guan et al., 2023). Additionally, XGBoost’s built-in regularization mechanism effectively reduces the risk of overfitting and enhances the model’s generalization ability, showing higher accuracy and sensitivity in various cancer studies (Zhang et al., 2020). This advantage allows XGBoost to effectively combine metabolic parameters (such as SUVmax) and anatomical features (such as the short diameter of lymph nodes) to significantly enhance the predictive accuracy for MLNM (Li et al., 2022). Therefore, this study not only demonstrates the superior performance of XGBoost but also provides new possibilities for individualized cancer treatment and prognostic prediction in the future (Guan et al., 2023; Yuan et al., 2022).

The study does have certain limitations. Owing to limitations in case availability, external validation of the TLPC model was not conducted. Prospective validation will be pursued as additional data are accumulated from our institution. Additionally, the use of 3D measurement tools for manual delineation of the VOI for the primary tumor and lymph nodes, while a well-established and validated approach, is time-consuming and subject to variability due to human operation. This limitation is inherent to manual delineation, especially when cross-institutional protocols differ. Such inter-operator variability may affect feature reproducibility and consistency across institutions, especially when delineation protocols or reconstruction parameters differ. Future work will incorporate semi-automated and deep learning–assisted segmentation methods to improve reproducibility and reduce operator dependence. Some patients in the retrospective study may not have had all required data collected. Nonetheless, this study included a relatively large number of patients and, for the first time, established a highest-performing predictive model for MLNM based on the radiological characteristics of both tumors and lymph nodes along with the clinical features of patients.

Although this study included a relatively large cohort, external validation in an independent population is still lacking, which may limit the generalizability of our findings. A prospective, multi-center external validation study is planned to further assess the robustness and clinical applicability of the TLPC model. Future validation efforts will focus on evaluating model calibration, discrimination, and decision curve performance across different imaging platforms and clinical settings, ensuring that the model can be reliably implemented in diverse real-world practice.

Furthermore, only 403 of the 1,149 resected patients underwent preoperative PET-CT, reflecting multidisciplinary team (MDT)-based referral rather than routine imaging for all surgical candidates. This selective acquisition likely enriched the cohort with patients having higher clinical suspicion of mediastinal involvement, potentially introducing spectrum or selection bias. Although stratified sampling, probability calibration, and prevalence-adjusted sensitivity analyses were performed to minimize these effects, residual bias cannot be completely excluded. Therefore, external validation in cohorts where PET-CT is routinely performed will be essential to confirm the model’s calibration and generalizability across different clinical settings.

## Supplemental Information

10.7717/peerj.20788/supp-1 Supplemental Information 1 Python code for machine learning model development and evaluation.

10.7717/peerj.20788/supp-2 Supplemental Information 2 Anonymized dataset containing clinical, pathological, and PET-CT imaging variables used in model construction and validation.

10.7717/peerj.20788/supp-3 Supplemental Information 3 Codebook.

10.7717/peerj.20788/supp-4 Supplemental Information 4 Acronyms.

10.7717/peerj.20788/supp-5 Supplemental Information 5 STROBE checklist.

## Additional Information and Declarations

### Data Availability

The following information was supplied regarding data availability:

The raw measurements are available in the Supplemental Files.
