# Supplementary material for: Machine learning-driven PET-CT and clinical pathology model for predicting mediastinal lymph node metastasis in non-small cell lung cancer: a retrospective cohort study
Source: PeerJ. 2026 Feb 3;14:e20788. doi: 10.7717/peerj.20788 (PMC12880095; doi:10.7717/peerj.20788)
Supplement: Supplemental Information 3 [file peerj-14-20788-s003.docx]

**Gender**: Male＝1 Female＝2

**Procedure**: 1＝Lobectomy　2＝Sublobectomy　3＝Pneumonectomy 4=EBUS-EBNA

**Lobar distribution of tumour**: 1=RUL 2=RML 3=RLL 4=LUL 5=LLL

**Operative approach**: thoracotomy=1　video-assistant thoracoscopic surgery，VATS=2 Endobronchial ultrasound guided tranbronchial needle aspiration EBUS-TBNA=3

**Smoking**:Yes=1 No=0

**Alcohol**:Yes=1 No=0

**Hypertension**:Yes=1 No=0

**Diabetes**:Yes=1 No=0

**Coronary heart disease**:Yes=1 No=0

**Nodule type** :mGGO=2／SN=3

**Spiculation sign**:Yes=1 No=0

**Lobulation sign**:Yes=1 No=0

**Bronchus encapsulated air sign**:Yes=1 No=0

**Cavity**:Yes=1 No=0

**Calcification**:Yes=1 No=0

**Bronchial cut-off sign**:Yes=1 No=0

**Margin**: Clear=0 Unclear=1

**Pleural indentation**:Yes=1 No=0

**History of lung disease**:Yes=1 No=0

**Histology of primary tumor** :adenocarcinoma=1　squamous cell carcinoma=2　adeno-squamous carcinoma=3　other=4

**Histologic subtype**:non- adenocarcinoma＝０　Acinous predominant adenocarcinoma＝１　Papillary predominant adenocarcinoma＝２　Micropapillary predominant adenocarcinoma＝３　Solid predominant adenocarcinoma＝４　other＝５ =Adenocarcinoma in situ Microinvasive adenocarcinoma Invasive mucinous adenocarcinoma

**N1 metastasis**:Yes=1 No=0

**Perineural invasion**:Yes=1 No=0

**Vascular invasion**:Yes=1 No=0

**Visceral pleural invasion**:Yes=1 No=0

**Tumor spread through air space STAS**:Yes=1 No=0

**T stage**: T1=1 T2=2 T3=3 T4=4

**N stage**: N2a1=1　N2a2=2　N2b1=3 N2b2=4　N1=5 N0=6

N1(+)=1 N1(-)=2
