# Supplementary material for: Machine learning-driven PET-CT and clinical pathology model for predicting mediastinal lymph node metastasis in non-small cell lung cancer: a retrospective cohort study
Source: PeerJ. 2026 Feb 3;14:e20788. doi: 10.7717/peerj.20788 (PMC12880095; doi:10.7717/peerj.20788)
Supplement: Supplemental Information 4 [file peerj-14-20788-s004.docx]

| **Acronym** | **Full Term** | **Definition / Unit** |
| --- | --- | --- |
| SUVmax | Maximum Standardized Uptake Value | Highest FDG uptake in ROI |
| SUVavg | Average Standardized Uptake Value | Mean FDG uptake in ROI |
| SULmax | Lean-body-mass–corrected SUVmax | SUV normalized by lean body mass |
| MTV | Metabolic Tumor Volume | Volume of metabolically active tumor (cm³) |
| TLG | Total Lesion Glycolysis | MTV × SUVmean |
| CTmax / CTavg | Maximum / Average CT attenuation value | Hounsfield units (HU) |
| CEA | Carcinoembryonic Antigen | Serum tumor marker (ng/mL) |
| SCCAg | Squamous Cell Carcinoma Antigen | Serum tumor marker (ng/mL) |
| CA199, CA125, CA724, CA153, CA242 | Carbohydrate Antigens | Tumor markers (U/mL) |
| FEV1 | Forced Expiratory Volume in 1 second | Pulmonary function parameter (L) |
| ProGRP | Pro-Gastrin-Releasing Peptide | Serum tumor marker (pg/mL) |
| AFP | Alpha-Fetoprotein | Serum tumor marker (ng/mL) |
| ESR | Erythrocyte Sedimentation Rate | mm/hour |
| D-dimer | Fibrin Degradation Product | mg/L |
